# Intradomain Confinement of Disulfides in the Folding of Two Consecutive Modules of the LDL Receptor

**DOI:** 10.1371/journal.pone.0132141

**Published:** 2015-07-13

**Authors:** Juan Martínez-Oliván, Hugo Fraga, Xabier Arias-Moreno, Salvador Ventura, Javier Sancho

**Affiliations:** 1 Biocomputation and Complex Systems Physics Institute (BIFI)-Joint Unit BIFI-IQFR(CSIC), Universidad de Zaragoza, Zaragoza, Spain; 2 Departamento de Bioquímica y Biología Molecular y Celular, Universidad de Zaragoza, Zaragoza, Spain; 3 Institut de Biotecnologia i Biomedicina and Departament de Bioquímica i Biologia Molecular, Universitat Autònoma de Barcelona, Bellaterra, Spain; 4 Departamento de Bioquimica, Faculdade de Medicina da Universidade do Porto, Porto, Portugal; The University of Queensland, AUSTRALIA

## Abstract

The LDL receptor internalizes circulating LDL and VLDL particles for degradation. Its extracellular binding domain contains ten (seven LA and three EGF) cysteine-rich modules, each bearing three disulfide bonds. Despite the enormous number of disulfide combinations possible, LDLR oxidative folding leads to a single native species with 30 unique intradomain disulfides. Previous folding studies of the LDLR have shown that non native disulfides are initially formed that lead to compact species. Accordingly, the folding of the LDLR has been described as a "coordinated nonvectorial” reaction, and it has been proposed that early compaction funnels the reaction toward the native structure. Here we analyze the oxidative folding of LA4 and LA5, the modules critical for ApoE binding, isolated and in the LA45 tandem. Compared to LA5, LA4 folding is slow and inefficient, resembling that of LA5 disease-linked mutants. Without Ca^++^, it leads to a mixture of many two-disulfide scrambled species and, with Ca^++^, to the native form plus two three-disulfide intermediates. The folding of the LA45 tandem seems to recapitulate that of the individual repeats. Importantly, although the folding of the LA45 tandem takes place through formation of scrambled isomers, no interdomain disulfides are detected, i.e. the two adjacent modules fold independently without the assistance of interdomain covalent interactions. Reduction of incredibly large disulfide combinatorial spaces, such as that in the LDLR, by intradomain confinement of disulfide bond formation might be also essential for the efficient folding of other homologous disulfide-rich receptors.

## Introduction

Disulfide bonds are key structural determinants of many extracellular and cell surface proteins. They have been known for long time to play an important role in protein conformational stability [1] and, more recently, to act as switches for protein function [2, 3]. In medium and large proteins, disulfide linkage constitutes an effective, but non-essential, strategy to increase stability. Small proteins, in contrast, tend to heavily rely on disulfides and/or cation binding to compensate for the small size of their hydrophobic cores [4]. Small disulfide-rich proteins are versatile and can perform a wide range of biological functions, acting as hormones, growth factors, proteases inhibitors, toxins and structural or ligand-binding domains within larger proteins [5].

**Fig 1:**
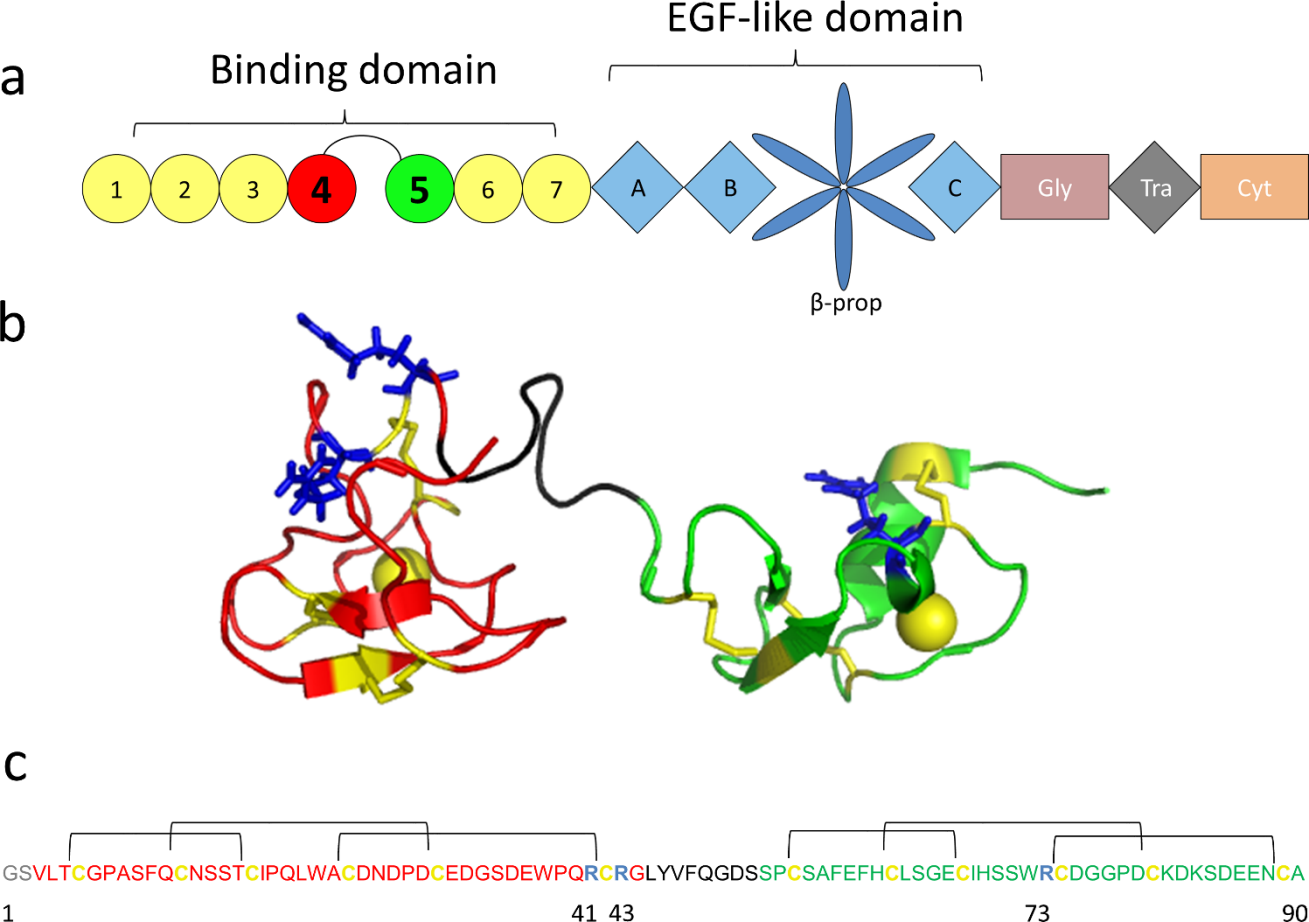
The LDL receptor and the LA45 pair of its lipoprotein binding domain. **(a)** Schematic representation of the LDL receptor. The LA domains (circles) are numbered from 1 to 7. Domains LA4 (in red) and LA5 (in green) are highlighted. The epidermal grow factor motifs (A, B and C) are shown as blue squares and the β-propeller motif is represented as a 6-bladed blue flower. The glycosylated (Gly; brown rectangle), transmembrane (Tra; black square) and cytoplasmatic (Cyt; orange rectangle) domains are also shown. **(b)** Representation of LA45 (PDB:2LGP) using Pymol. The LA4 residues are marked in red and those of LA5 are shown in green. The linker between LA4 and LA5 is shown in black and arginines are highlighted in dark blue. Cysteines and calcium ions are shown in yellow and native disulfide bonds are shown as yellow sticks. ARG-C proteinase cleavage sites are present in the C-terminal of arginine residues. **(c)** Amino acid sequence of recombinant LA45. The first two residues (GS; in grey) were added in the cloning and are not present in wild type LA45. All other residues are presented in the same colors used in the structural representation. Disulfide bonds are shown as black lines.

Certain cell surface receptors bank on the presence of disulfide-rich domain repeats to bind their molecular targets. An example of this architecture is found in the low-density lipoprotein receptor (LDLR) gene family, encompassing structurally related receptors involved in cholesterol homeostasis and cell signaling, among other functions [6–8]. One of the smallest members of the family is the LDLR itself, a modular receptor responsible for binding circulating LDL and VLDL remnants for cellular degradation [9–11]. When bound to the cell membrane, most of the LDLR is extracellular [12]. From the N to the C terminus, the LDLR contains seven small ligand binding repeats (LA1 to LA7), two epidermal growth factor-like domains (EGFA and EGFB), one β-propeller domain, one additional EGF domain (EGFC), one glycosylated domain, a transmembrane helix and one small cytoplasmic domain (Fig 1A). The seven LA repeats [13] are small disulfide-rich domains of about 40 residues. They contain three intradomain disulfide bonds and a bound Ca^++^ ion [12, 14–18]. Point mutations in LA repeats are a frequent cause of Familial Hypercholesterolemia (FH). LA repeats (Fig 1B) show high structural independence [19–21] and are connected by small flexible 4–6 residue linkers, an exception being the longer 12-residue connector between LA4 and LA5 [12, 19]. Similarly to the LA repeats, each of the three EGF-like domains contains three intradomain disulfide bonds [22, 23].

The oxidative folding of the LDLR into a single species with 30 unique disulfides appears challenging because its 60 cysteine residues can, in principle, pair in ~3 x 10^40^ different forms. Such huge number is reminiscent of the Levinthal’s paradox [24] that lead to the conclusion that newly synthesized polypeptides could not randomly search among all possible conformations but should follow folding pathways permitting them to arrive to the native conformation in times compatible with life. While it is clear that proteins such as the LDLR, containing dozens of disulfide bonds cannot randomly attain the native conformation, it is not evident how their efficient folding is achieved. Non-disulfide containing proteins have been proposed to experience an initial hydrophobic collapse that could drastically reduce the conformational entropy of the polypeptide, making the subsequent conformational search fast, and one possibility is that disulfide-rich large proteins experience an analogous initial collapse driven by non-native disulfide formation. However, although such a collapse would reduce the conformational entropy because the polypeptide would be confined to a smaller volume, the entropic problem associated to the enormous number of possible disulfide combinations would remain. An alternative possibility for disulfide-rich large modular proteins such as LDLR, where most disulfide bonds occur inside defined domains, is that formation of disulfides occurs in a co-translational manner so that as soon as the most N-terminal disulfide containing domain arrives to the endoplasmic reticulum it forms its own disulfides, followed by sequential, non-competing formation of the disulfides in the following domain and so forth. In this way, the combinatorial problem posed by the enormous number of possible disulfide would fade. A combination of those two extreme mechanisms cannot be discarded.

Pioneering work on the oxidative folding of the LDLR [25, 26] found that, in the early stages of folding, the receptor forms interdomain disulfides between distant domains and that the native disulfide connectivity can only be attained in the presence of Ca^++^ ions. In the LDLR, two LA modules, LA4 and LA5, have been identified as the more relevant ones for the binding and release of lipoproteins [13, 27–29]. In previous work we have analyzed the oxidative folding pathway of LA5 and shown that, in presence of Ca^++^, the native form is spontaneously obtained [30]. Here we have investigated the oxidative folding of LA4, a less stable module with a significantly lower affinity for Ca^++^ [19, 31]. Our analysis indicates that LA4 folding is reminiscent of that of LA5 mutants associated to Familial Hypercholesterolemia, which are characterized by deficient Ca^++^ binding [30]. Trying to understand how the modular structure of the receptor affects oxidative folding we have analyzed the folding of the LA45 tandem. Contrary to the notion that interdomain disulfides could accelerate folding, no such linkages have been observed between modules LA4 and LR5.

## Materials and Methods

### Expression and purification of the LA constructs

Recombinant LA4, LA5 and LA45 were cloned as described [31, 32] and expressed fused to glutathione S-transferase (GST). The purification, as described in [29–32], includes glutathione affinity chromatography, thrombin cleavage plus removal of GST, oxidative refolding by redox dialysis, and RP-HPLC on a preparative C4 column. Purity >99% was determined by SDS-PAGE and MALDI-TOF MS. RP-HPLC elution profiles as well as calorimetric and spectroscopic analysis of Ca^++^ binding to the isolated recombinant LA´s (LA4, LA5 and LA45) [31] compared to the data obtained for the native forms of the LA45 domain for which the structure was solved by NMR [19] showed that our LA domains were also in native conformation and, accordingly, they were used as a control in RP-HPLC experiments. Lyophilized protein was stored at -20°C. The theoretical extinction coefficients [33] were used to estimate the concentration of the LA domains.

### Oxidative folding of LA4 and LA45

Native forms of LA4 or LA45 were reduced by incubation in Tris-HCl buffer, pH 8.4, 50 mM dithiothreitol (DTT; Sigma-Aldrich, St. Louis, Missouri) containing 6 M guanidine thiocyanate (GdnSCN; Sigma-Aldrich) at 23°C for at least 4 hours. Then, the samples were passed through a PD 10 column Sephadex G 25 M (GE Healthcare, Fairfield, Connecticut) to exchange the buffer and initiate the folding. Different experiments were carried out in Tris-HCl buffer, pH 8.4, including either 0.1 mM EDTA or different concentrations of CaCl_2_ (from 1 to 1000 mM). LA4 or LA45 concentrations were 0.5 mg/ml in all cases, and different experiments were performed in absence or presence of 0.25 mM 2-mercaptoethanol (BME), 0.5 mM oxidized glutathione (GSSG, Sigma-Aldrich) or 150 mM NaCl and 2 μM protein disulfide-isomerase (PDI, Sigma-Aldrich). Some experiments were additionally performed at 4, 15 and 37°C. To stop the folding reaction, 25-μl samples were removed at different times and acidified with 4% TFA. Samples were then analyzed by RP-HPLC using a 60 minutes 15–39% water/acetonitrile linear gradient with TFA 0.1% in a 4.6-mm Jupiter C4 column (Phenomenex, Torrance, California). At the same time points, 1-μl samples were derivatized with 0.5 μl of 0.3 M vinylpiridine (Sigma-Aldrich) for 45 min at room temperature in darkness and then diluted to 15 μl with 0.1% TFA and analyzed by mass spectrometry in a MALDI-TOF ultraFlextreme (Bruker Daltonik GmbH, Billerica, Massachusetts) in reflectron mode, under 25 kv, using external calibrators. Samples were diluted 1:1 with matrix solutions (10 mg/ml of 2.6-dihydroxyacetophenone dissolved in 30% acetonitrile containing 20 mM ammonium citrate, pH 5.5) and spotted on a ground steel plate. Analysis of the spectra was performed with the Flex Analysis 3.4 program (Bruker Daltonik GmbH).

### Analysis of interdomain disulfide bond formation during LA45 folding

During the oxidative folding of LA45 in presence of either 10 mM CaCl_2_ or 0.1 mM EDTA, aliquots were removed at different times and derivatizated as explained in the previous section, without acidification. Derivatizated samples were then cleaved with Arg-C proteinase (Sigma-Aldrich) in a 10:1 w/w ratio at 37°C for 8 hours. The digested solutions were diluted 10 times with 0.1% TFA and analyzed by MALDI-TOF as described above.

### Foldability of LA4 intermediates previously obtained in absence of calcium

Reduced LA4 or LA45 were allowed to fold for 24 hours in 0.1 M Tris-HCl, pH 8.4 with 0.1 mM EDTA. Then, 1 to 1000 mM CaCl_2_ was added to the samples in absence or presence of 0.25 mM BME or 0.5 mM GSSG. After 48 h, folding was stopped by adding 4% TFA and the samples were analyzed by RP-HPLC.

### Stop and go folding of the LA4 scrambled intermediate, and disulfide scrambling of the LA4 native form

After oxidative folding of LA4 in presence of 10 mM CaCl_2_, the major LA4 scrambled intermediate (Xa4) was isolated by RP-HPLC as described above for the purification of native LA4. In order to better separate native and scrambled LA4 forms, a 150-minute, 15–39% linear gradient (water/acetonitrile with 0.1% TFA) was run. The scrambled form was lyophilized, dissolved in 0.1 M Tris-HCl, pH 8.4 and different CaCl_2_ concentrations from 1 to 1000 mM in absence or presence of 0.25 mM BME, and incubated at 23°C. Similarly, native LA4 was dissolved in 0.1 M Tris-HCl, pH 8.4 with either 0.1 mM EDTA or 1 to 10 mM CaCl_2_ in absence or presence of 0.25 mM BME. Aliquots at different times were trapped by acidification and analyzed by RP-HPLC.

## Results

### Oxidative folding of LA4 yields a mixture of native and scrambled forms

**Fig 2:**
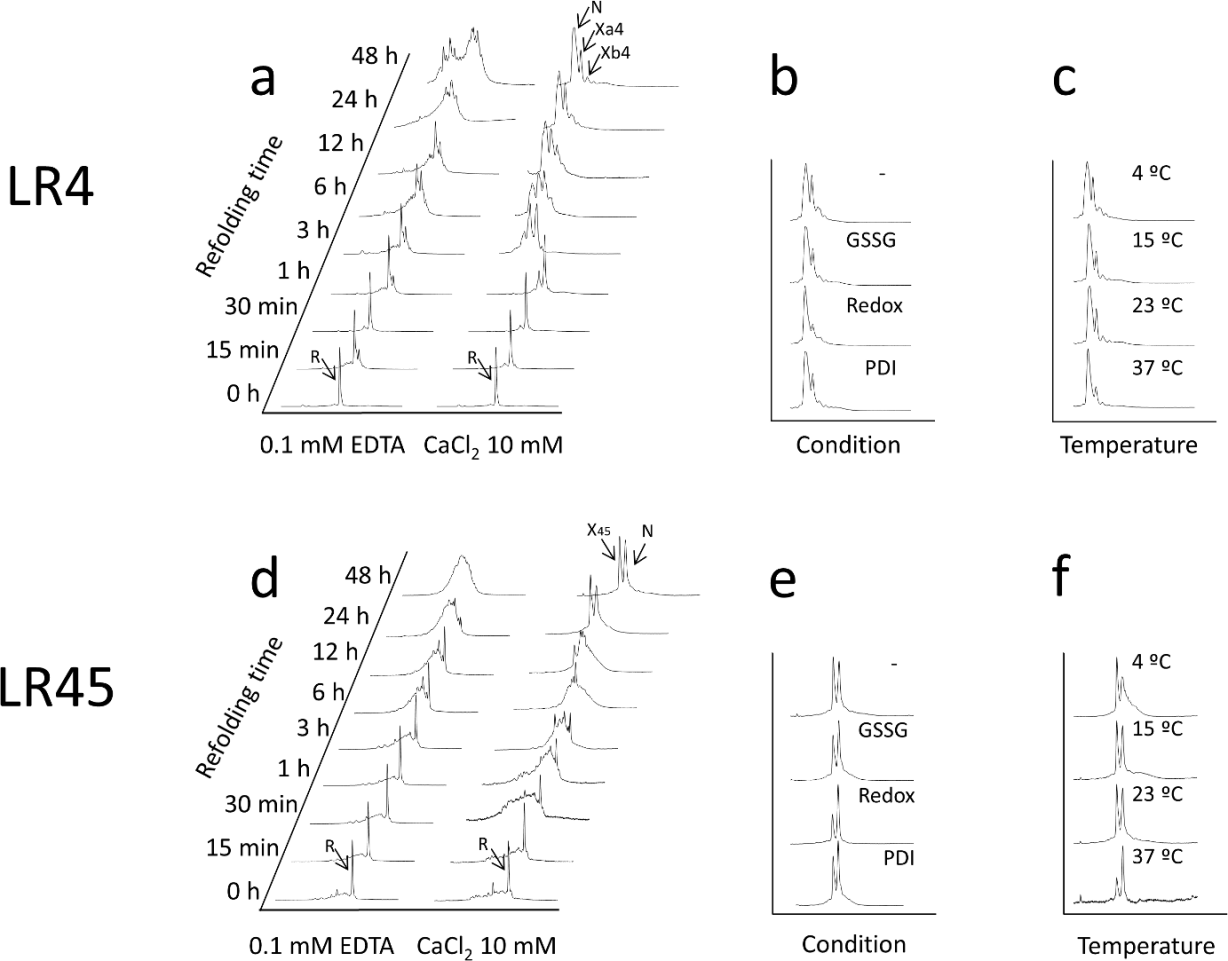
Oxidative folding of LA4 and LA45. **(a, d)** RP-HPLC traces of acid trapped intermediates in the folding of reduced LA4 (*a*) or LA45 (*d*) in Tris-HCl buffer, pH 8.4, with either 0.1 mM EDTA or 10 mM CaCl_2_. In panel *a*, N, Xa4 and X4b correspond, respectively, to native LA4, a major and a minor LA4 scrambled isomers, while R corresponds to the fully reduced form. In panel *d*, N and X45 correspond to native LA45 and a major scrambled isomer, respectively, while R corresponds to reduced LA45. **(b, e)** Chromatograms after 48 hours folding of LA4 (*b*) or LA45 (*e*) in Tris-HCl buffer, pH 8.4 with 10 mM CaCl_2_ and either 0.25 mM 2-mercaptoethanol, 0.5 mM GSSG (GSSG), 150 mM NaCl and 2 μm PDI (PDI) or no additives (-). **(c, f)** Chromatograms after 48 hours folding of LA4 (*c*) or LA45 (*f*) in Tris-HCl buffer, pH 8.4 with 10 mM CaCl_2_ at 4, 15, 23 or 37°C.

**Fig 3:**
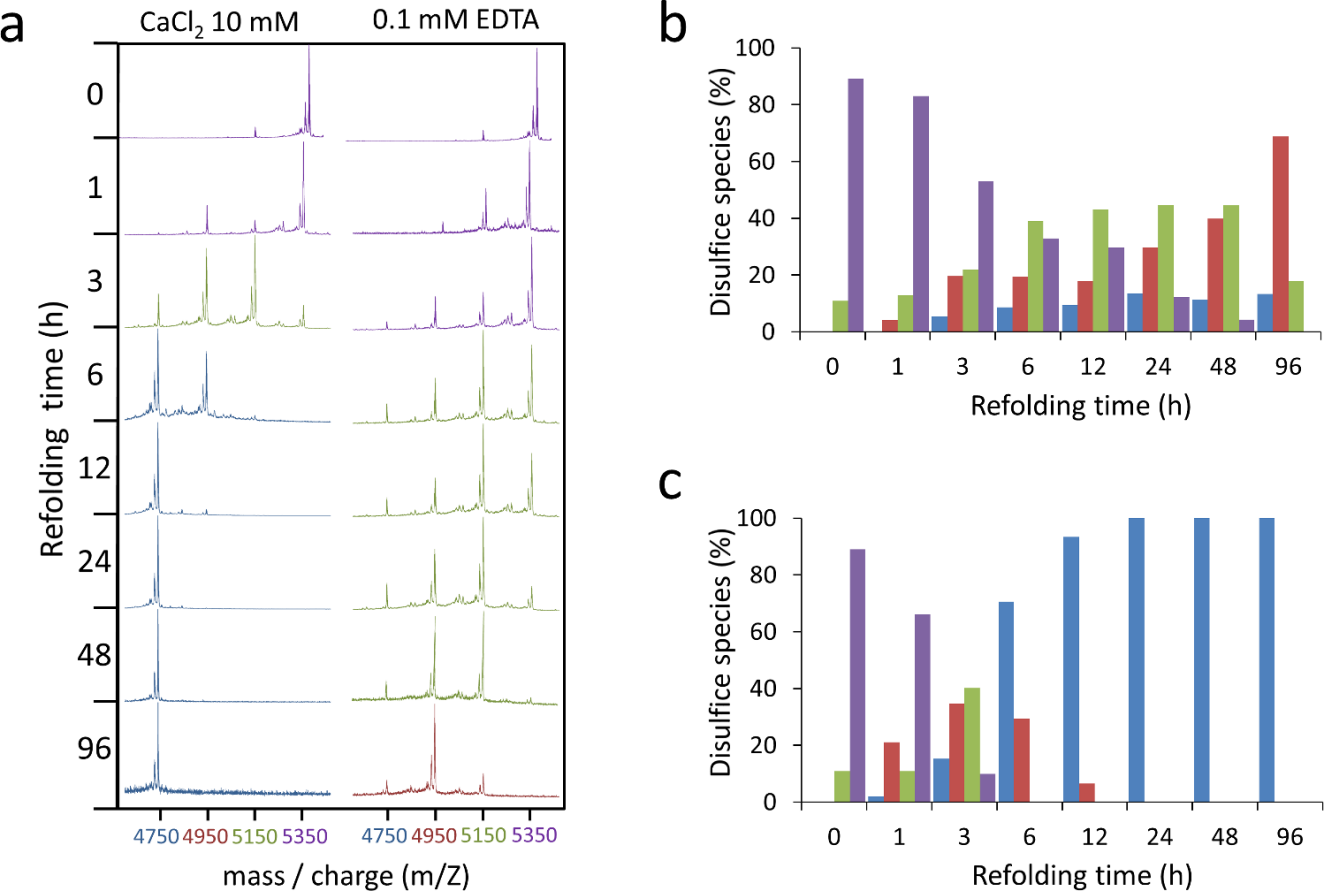
Analysis of disulfide species in LA4 folding. **(a)** MALDI-TOF spectra of derivatizated LA4 intermediates along LA4 folding in Tris-HCl buffer, pH 8.4 with either 0.1 mM EDTA or 10 mM CaCl_2_. Expected mass of LA4 species with 3, 2, 1 or none disulfide bonds (3S, 2S, 1S or 0S) are, respectively 4744, 4952, 5160 and 5368 Daltons. Spectra where the major species are 3S are shown in blue, red is used for 2S, green for 1S and purple for 0S. **(b, c)** Percentages of disulfide species found during LA4 folding in Tris-HCl buffer, pH 8.4 with either 0.1 mM EDTA (*b*) or 10 mM CaCl_2_ (*c*). Percentages were calculated based on relative intensities of the peaks in the MALDI-TOF spectra. 3S, 2S 1S and 0S species are shown respectively in blue, red, green and purple.

**Fig 4:**
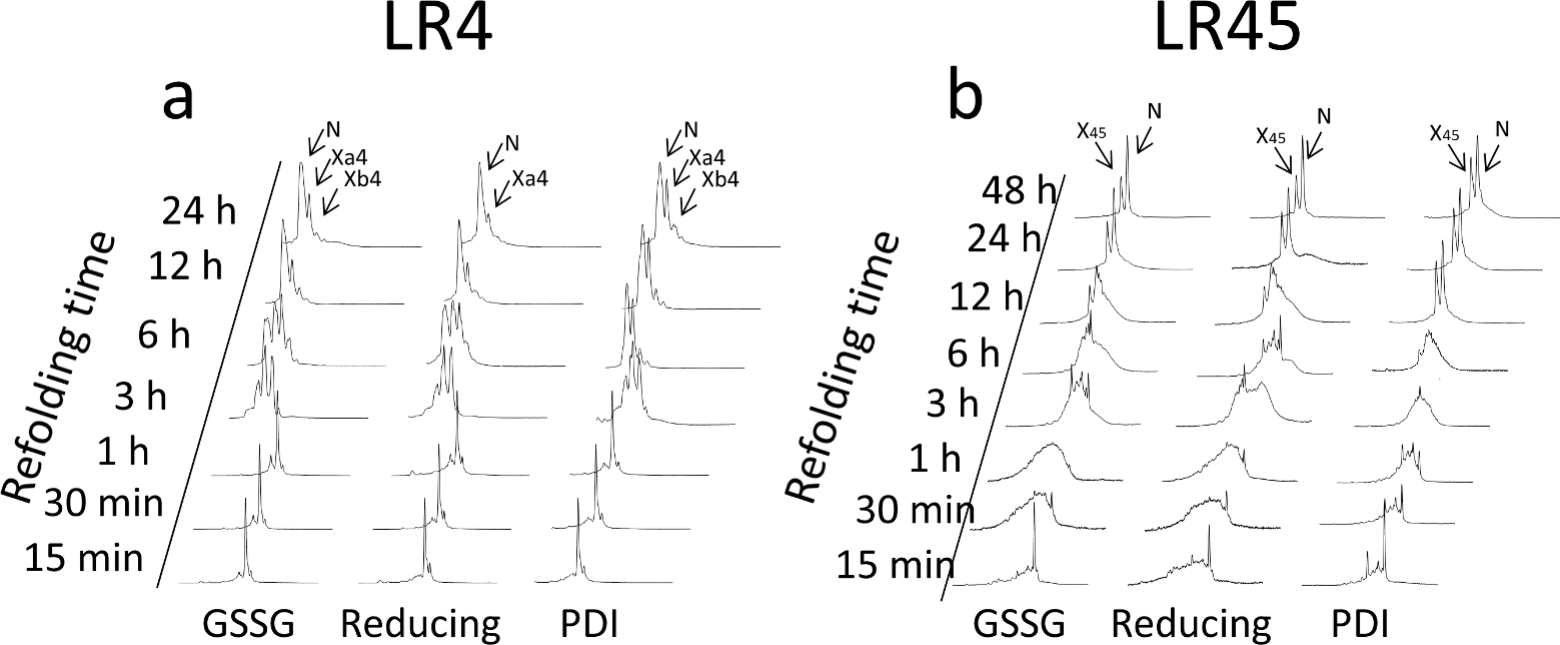
Oxidative folding of LA4 and LA45. **(a,b)** RP-HPLC traces of acid trapped intermediates in the folding of reduced LA4 (*a*) or LA45 (*b*) inTris-HCl buffer, pH 8.4 with 10 mM CaCl_2_ and either 0.25 mM BME, 0.5 mM GSSG (GSSG) or 150 mM NaCl and 2 μm PDI (PDI). In panel *a*, N, Xa4 and X4b correspond respectively to native LA4, and its major and minor scrambled isomers. R corresponds to the fully reduced form of LA4. In panel *b*, N and X45 correspond to native LA45 and the major scrambled isomer of LA45, and R corresponds to reduced LA45.

In absence of Ca^++^, the oxidative folding of reduced LA4 at 23°C produced, after 48 h, a complex mixture of isomers (Fig 2A) containing one, two and three disulfide bonds (Fig 3A). In contrast, the same reaction in presence of Ca^++^ yielded, in 24 hours, just three LA4 isomers, as previously observed in similar conditions [19, 34]. The earliest eluting species was native LA4 and the two others, Xa4 and Xb4, were scrambled isomers containing three disulfide bonds (Figs 2A and 3A). When the folding was performed in presence of Ca^++^ and 2-mercaptoethanol (BME), the kinetics were not significantly accelerated but the final population was largely enriched in native LA4 (Figs 2B and 4A). Folding in presence of Ca^++^ plus either oxidized glutathione (GSSG) or protein disulfide-isomerase (PDI) (Figs 2B and 4A) did not modify the early stages or the final populations of native LA4, Xa4 and Xb4. However, PDI accelerated the late stages of the reaction and the equilibrium was reached in 6 hours (Fig 4A). Oxidative folding in presence of Ca^++^ at different temperatures revealed that the population of native LA4 increased at the expense of the scrambled isomers when the temperature was raised (Fig 2C).

### LA45 oxidative folding occurs without interdomain disulfide formation

**Fig 5:**
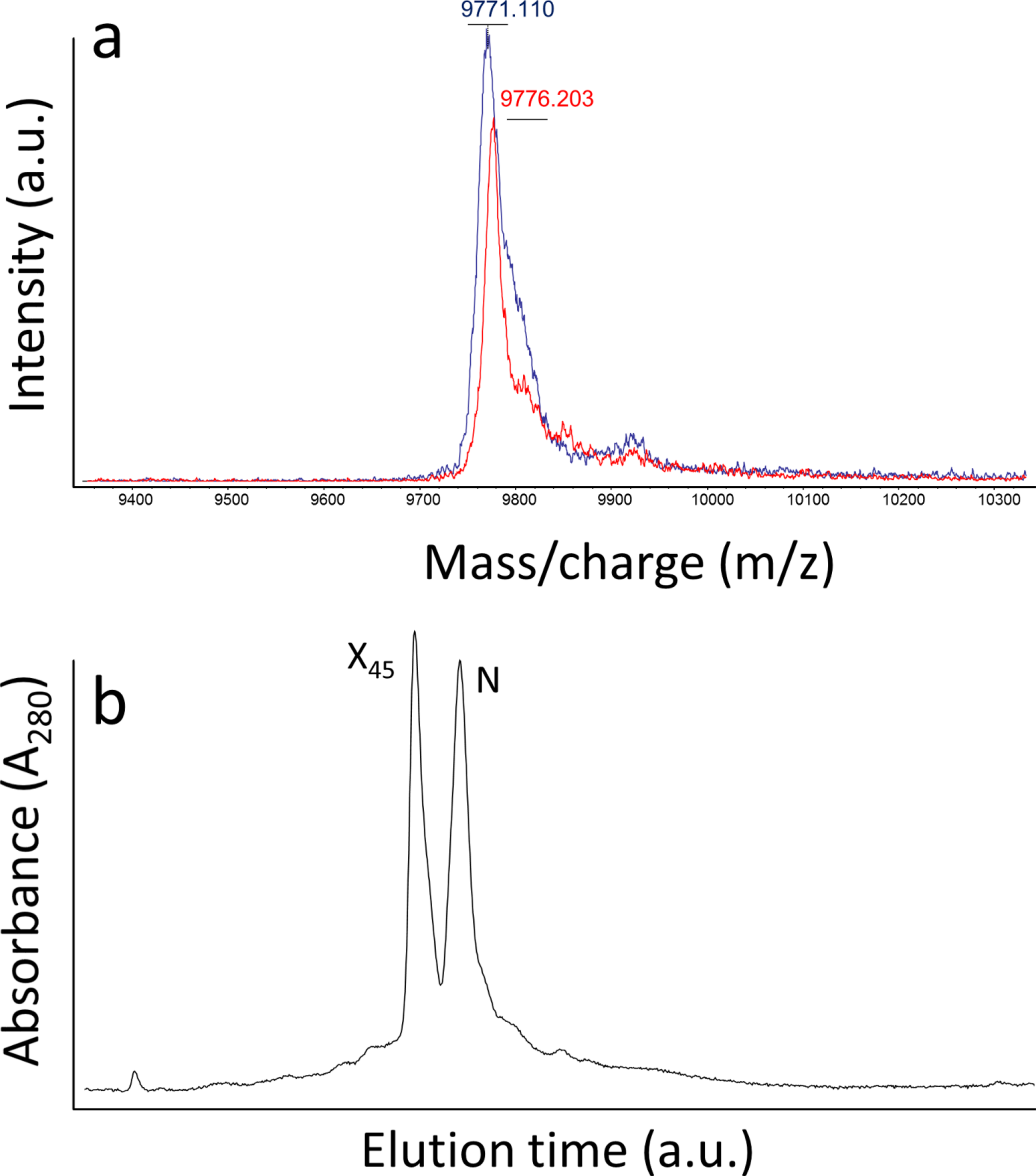
Analysis of disulfide species after LA45 folding. **(a)** Red and blue plots correspond to replicate MALDI-TOF spectra of derivatizated intermediates after 48 hours folding of reduced LA45 in Tris-HCl buffer, pH 8.4 with 10 mM CaCl_2_. Expected mass is 9782 daltons for species with 6 disulfide bonds and 208 daltons more per disulfide bond unformed. There is only one peak corresponding to fully oxidized LA45 forms, without free cysteines. **(b)** RP-HPLC traces of the same LA45 intermediates analyzed in *a*. N and X45 correspond to native LA45 and the major scrambled isomer of LA45, respectively.

The oxidative folding reaction of LA45 in the absence of thiol catalysts results in the formation of two fully oxidized isomers, each containing six disulfide bonds: native LA45 and a scrambled form (X45) (Figs 2D and 5). The dependence of the efficiency of the folding reaction on the temperature (Fig 2F) and on the presence of thiols (Fig 2E) indicates that, similar to Xa4, X45 constitutes a highly stable intermediate whose energy barrier relative to LA45 decreases at higher temperatures or when reshuffling is allowed (Fig 2E and 2F). The fact that LA4 and LA5 fold independently, together with the higher conformational stability of LA5 in the presence of Ca^++^ and its more efficient folding reaction rendering a single native form suggest that X45 could consist of LA5 in its native conformation and a scrambled LA4 form, which would explain why the folding properties of X45 and Xa4 are similar.

**Fig 6:**
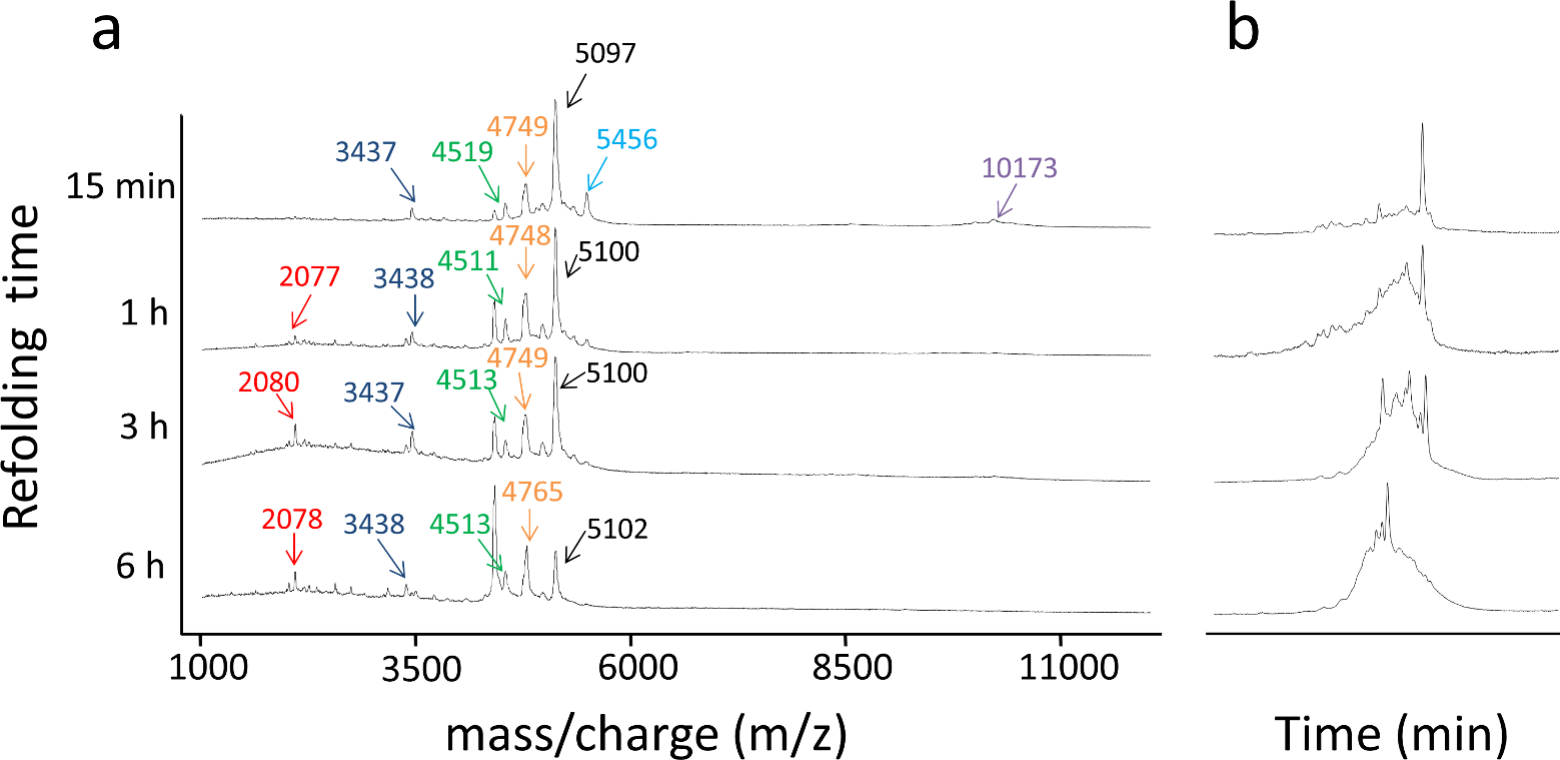
Analysis of interdomain disulfide bonds formation in LA45. **(a)** MALDI-TOF spectra of LA45 folding intermediate fragments obtained after derivatization and cleavage with ARG-C proteinase. Red, fragment 74–90 0S; Dark Blue, fragment 44–73 1S; Green, fragment 1–41 2S; Orange 1–41 1S; Black 1–41 + 42–43 1S or, less probable, 44–73 + 74–90 3S, Light Blue, 42–43 + 44–73 + 74–90 3S; Purple, 1–41 + 42–43 + 44–73 + 74–90 4S or 5S. All fragments correspond to fragments without interdomain formation except the small signals detected after 15 min at 5456 (Light Blue) and 10173 daltons (purple). ±0,5–1% SD is expected in the mass data. The fragments that appear in this figure are highlighted in bold type in Tables 1 and 2. **(b)** RP-HPLC traces of the same LA45 intermediates analyzed in *a*.

To elucidate whether transient interdomain disulfide bonds were formed during the folding of LA45, derivatized intermediates were cleaved with ARG-C proteinase and the peptide fragments analyzed by MALDI-TOF. LA45 contains three ARG-C proteinase cleavage sites, at residues 41, 43 and 73, which should lead to four peptide fragments encompassing residues 1–41, 42–43, 44–73 and 74–90 of the recombinant LA45 used (Fig 1C) if no disulfide bonds were present or to a combination of fragments 1–41, 42–43, 1–43, 44–73, 74–90 and 44–90 if only intradomain disulfide bonds, native or otherwise, were present. Therefore, in absence of interdomain disulfide bonds, no fragments of MM > 5600 Daltons are expected after cleavage with ARG-C proteinase (Table 1). In contrast, any folding intermediate bearing interdomain disulfide bonds involving cysteines I, II, III, IV or V in LA4 and any cysteine in LA5 is expected to produce fragments bigger than 6400 Daltons after derivatization and digestion (Table 2). However, no fragments with MM >6000 Daltons were detected during the oxidative folding of LA45 in presence of 10 mM Ca^++^ (Fig 6) or in its absence (not shown). On the other hand, folding intermediates bearing interdomain disulfide bonds involving cysteine VI in LA4 and any cysteine in LA5 are expected to produce either fragments bigger than 5500 Daltons or fragments at 3601 or 3809 Daltons (1S or 2S 42–73) or at 2037 or 2245 daltons (1S or 2S 42–43+74–90), which were not observed either (Table 1B). The most intense fragments detected between 4000 and 5400 Daltons corresponded to the expected 1–43 and 44–90 fragments in different oxidative forms. Fragments 44–73 and 74–90 were also seen as lower intensity bands around 3400 and 2100 Daltons. The small peaks at 5456 and 10173 observed in the earliest folding time analyzed might correspond either to traces of incompletely digested LA45 or to a very small population of interdomain disulfides. These results together indicate that no significant amount of LA4/LA5 interdomain disulfide bonds were formed during the oxidative folding of LA45 *in vitro*.

**Table 1:** Expected mass of LA45 fragments with no interdomain disulfide bonds.

Fragment	Number of disulfide bonds			
	3	2	1	0
**1–41**	-	**4541**	**4749**	4957
**42–43**	-	-	-	359
**44–73**	-	-	**3454**	3662
**74–90**	-	-	1890	**2098**
**1–41 + 42–43**	4688	4896	**5104**	-
**44–73 + 74–90**	**5136**	5344	5552	-

Expected mass (Daltons) of fragments of derivatizated recombinant LA45 cleaved with ARG-C proteinase if no interdomain disulfide bonds are established between LA4 and LA5. Fragments observed in Fig 6 are highlighted in bold.

**Table 2:** Expected mass of LA45 fragments with interdomain disulfide bonds.

Fragment	Number of disulfide bonds					
	6	5	4	3	2	1
**1–41 + 44–73**	-	-	7787	7995	8203	8411
**1–41 + 74–90**	-	-	6431	6639	6847	7055
**1–41 + 42–43 + 44–73**	-	-	8142	8350	8558	-
**1–41 + 42–43 + 74–90**	-	-	6578	6782	6980	-
**1–41 + 44–73 + 74–90**	-	9677	9885	10093	10301	-
**42–43 + 44–73**	-	-	-	-	3601	3809
**42–43 + 74–90**	-	-	-	-	2037	2245
**42–43 + 44–73 + 74–90**	-	-	-	**5491**	5699	-
**1–41 + 42–43 + 44–73 + 74–90**	9824	**10032**	**10240**	10448	-	-

Expected mass (Daltons) of fragments of derivatizated recombinant LA45 cleaved with ARG-C proteinase if interdomain disulfide bonds are established between LA4 and LA5. Fragments observed in Fig 6 are highlighted in bold.

### Ca^++^ drives misfolded LA4 and LA45 forms into the canonical folding route

**Fig 7:**
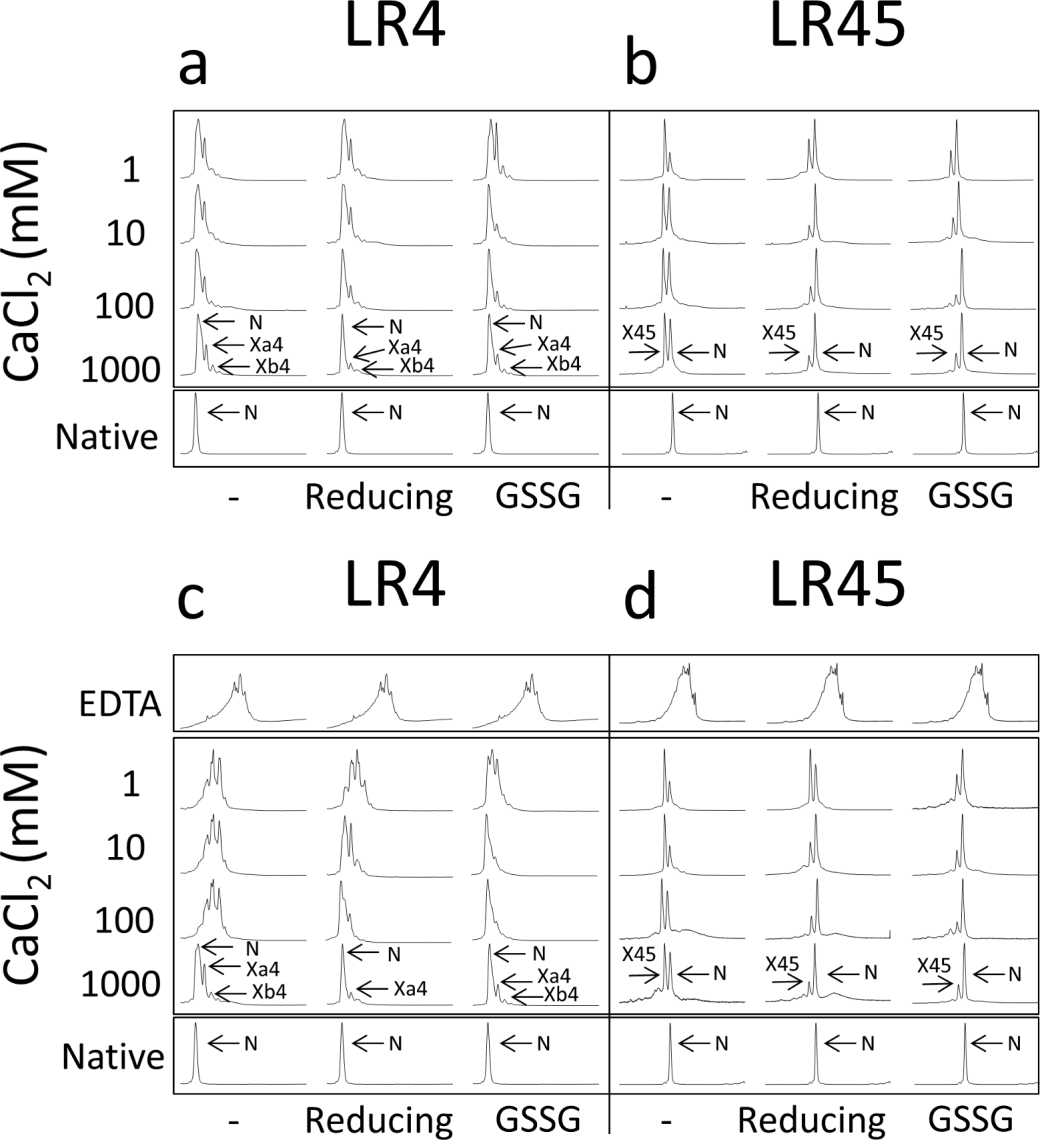
Reversibility of LA4 and LA45 intermediates formed in the absence of calcium. **(a,b)** RP-HPLC traces of LA4 (a) or LA45 (b) species formed after 48 hours folding of fully reduced LA4 (a) or LA45 (b) in Tris-HCl buffer, pH 8.4 with 1 to 1000 mM CaCl_2_ and either 0.25 mM 2-mercaptoethanol, 0.5 mM GSSG (GSSG) or no additives (-). The chromatograms of native LA4 (a) and native LA45 (b) are also shown in the lower row for comparison. **(c,d)** RP-HPLC traces of LA4 (c) or LA45 (d) species formed after 24 hours folding of fully reduced LA4 (c) or LA45 (d) in Tris-HCl buffer, pH 8.4 with 0.1 mM EDTA and then 48 hours in Tris-HCl buffer, pH 8.4 with 1 to 1000 mM CaCl_2_ and either 0.25 mM 2-BME, 0.5 mM GSSG (GSSG) or no additives (-). The chromatograms of native LA4 (c) and native LA45 (d) are also shown in the lower row for comparison. In the upper row, the chromatograms of LA4 intermediates after 24 hours folding of fully reduced LA4 (c) or LA45 (d) inTris-HCl buffer, pH 8.4 with 0.1 mM EDTA are shown for comparison.

Oxidative folding of LA4 or LA45 without Ca^++^ yielded a mixture of non-native forms. To determine whether Ca^++^ could assist those scrambled forms to fold properly, reduced LA4 or LA45 were allowed to fold for 24 hours in presence of 0.1 mM EDTA. Then, different concentrations of CaCl_2_ (from 1 to 1000 mM) were added, either alone or together with BME or GSSG, and the folding was allowed to continue for 48 additional hours. High Ca^++^ concentrations (1000 mM) suffice, in absence of redox catalysts, to yield the LA4 folding pattern (Fig 7C) dominated by the native form that was found when Ca^++^ was present from the beginning (Fig 7A). In presence of redox catalysts, lower Ca^++^ concentrations were needed (Fig 7A and 7C).

**Fig 8:**
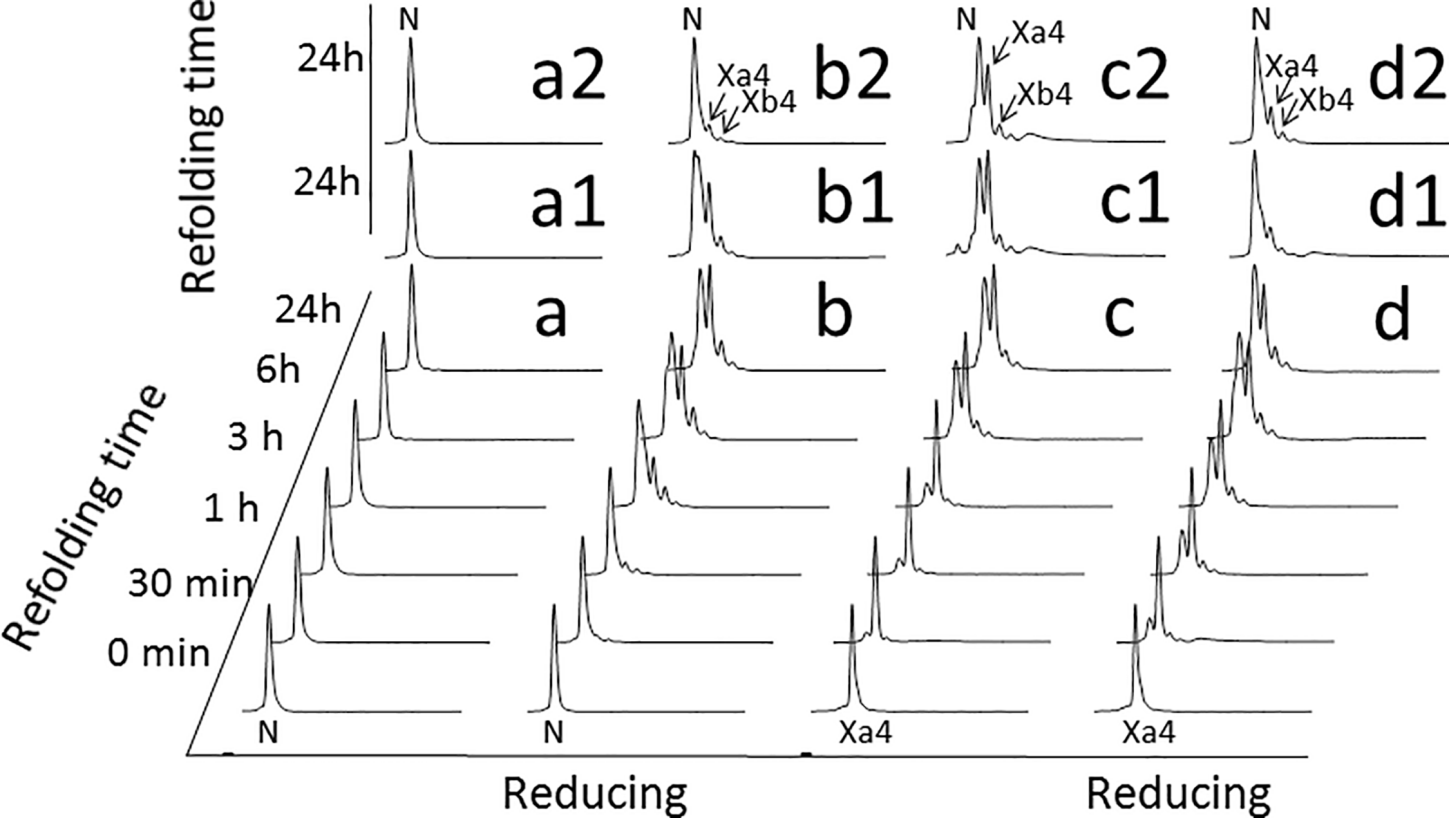
Stop and go of the LA4 scrambled isomer Xa4 and reshuffling of native LA4. **(a,b)** RP-HPLC of time course intermediates along the native LA4 reshuffling in Tris-HCl buffer, pH 8.4, 0.1 mM EDTA, without (*a*) or with (*b)* 0.25 mM 2-mercaptoethanol reducing agent. **(a1, a2)** LA4 isomers after 24 hours reshuffling of native LA4 in Tris-HCl buffer with 1 (*a1*) or 10 (*a2*) mM CaCl_2_. **(b1, b2)** LA4 isomers after 24 hours reshuffling of native LA4 in Tris-HCl buffer with 0.25 mM 2-mercaptoethanol and 1 (*b1*) or 10 (*b2*) mM CaCl_2_. **(c,d)** RP-HPLC of time course intermediates along the Xa4 scrambled isomer of LA4 stop and go in Tris-HCl buffer, pH 8.4, 10 mM CaCl_2_, without (*c*) or with (*d)* 0.25 mM 2-mercaptoethanol reducing agent. **(c1, c2)** LA4 isomers after 24 hours reshuffling of Xa4 in Tris-HCl buffer with 100 (*c1*) or 1000 (*c2*) mM CaCl_2_. (**d1, d2)** LA4 isomers after 24 hours reshuffling of Xa4 in Tris-HCl buffer with 0.25 mM 2-mercaptoethanol and 100 (*d1*) or 1000 (*d2*) mM CaCl_2_.

In the oxidative folding of LA45, the equilibrium between native LA45 and X45 at the end of the reaction in the absence of catalysts is dependent on the Ca^++^ concentration in the 1–10 mM range. In fact, X45 is the most populated species and therefore the most stable conformer at 1 mM calcium, which argues that the overall affinity of LA45 for Ca^++^ is low, a characteristic that can be unambiguously assigned to the LA4 repeat (Fig 7B and previous work [31]). However, the LA45/X45 ratio does not change in the 10–1000 mM calcium concentration range, reflecting a high kinetic barrier between them (Fig 7B). As for the Xa4/LA4 equilibrium, thiol agents lower the barrier and LA45 becomes the predominant species already at 10 mM Ca^++^, and additional indication that the inter-conversion between species involves reshuffling reactions (Fig 8B).

### The LA4 major scrambled isomer, Xa4, is transformed into the native form without reducing agents in presence of high Ca^++^ concentration

Isolated Xa4 was lyophilized, dissolved in buffer containing 10 mM Ca^++^, with or without 0.5 mM BME, and allowed to re-initiate folding (Fig 8C and 8D). Reshuffling of Xa4 took place in both absence or presence of thiol catalyst and some native LA4 and scrambled Xb4 were already detected after 30 minutes. After 24 hours, the three species were still present and the reaction appeared to have reached equilibrium. Higher Ca^++^ concentrations in presence of BME gave rise to higher populations of native LA4 (Fig 8D1 and 8D2).

On the other hand, native LA4 was dissolved in a buffer without Ca^++^ (with 0.1 mM EDTA). In the absence of thiol catalyst, no reshuffling was observed after 24 hours (Fig 8A). When BME was added, reshuffling could be observed before 1 hour and a mixture of native LA4, Xa4 and Xb4 was established after 24 hours (Fig 8B). When 1 mM Ca^++^ was additionally added, the mixture contained, after 24 hours, a higher proportion of native LA4 (Fig 8B1). When 10 mM Ca^++^ was added, the native LA4 peak largely dominated the chromatogram (Fig 8B2).

## Discussion

### Role of Calcium at the Packing and Consolidation Stages of LA4 oxidative folding

In presence of Ca^++^, denatured and reduced LA4 folds to the native structure through a sequential oxidation of cysteine residues that give rise to equilibrated ensembles of l-, 2-, and 3-disulfide conformers (Figs 2 and 3). None of the tested thiol catalysts accelerates the flow between reduced/unfolded and partially oxidized species or changes the apparent composition of these ensembles or promotes the population of a predominant meta-stable intermediate during the early stages of folding (Fig 4A). This indicates that, as previously shown for LA5 [33], the packing phase of LA4 essentially involves entropy restriction through non-specific pairing between free cysteines.

The first evident effect of Ca^++^ is an accelerated formation of 2-disulfide species at the first stages of the reaction (<1h) (Fig 3). In LA4, 7 acidic residues are concentrated in a 12-residue sequence stretch [35], the cation may be needed to prevent strong local repulsion and/or to promote a collapse that might allow subsequent oxidation of approximated cysteines. Accordingly, Ca^++^ causes a strong reduction in the heterogeneity of the 2-disulfide ensemble leading to the formation of a restricted set of intermediates (Fig 2A). Ca^++^ coordination at this stage would funnel consolidation toward the formation of 3-disulfide species, since it would promote a strong restriction in the conformational search to oxidize native disulfide bonds and thus a significant increase in the folding rate. The dramatic impact that Ca^++^ exerts in the LA4 folding pathway is best observed when comparing the last stages of folding in the presence and absence of the cation. Without Ca^++^ the reaction leads to the formation of a very heterogeneous ensemble, strongly dominated by 2-disulfide species (Fig 2A), which implies that the absence of the cation essentially impedes the consolidation step.

### The oxidative folding landscape of LA4 resembles that of FH-linked LA5 variants

Despite the mechanistic similitude at the packing and consolidation stages of LA4 and LA5 repeats, the folding landscapes of these two structurally homologous domains exhibit important differences. In absence of Ca^++^, LA5 oxidative folding ends up with formation of fully oxidized, scrambled isomers with accumulation of a particular scrambled form (Xa5) accounting for more than 60% of the protein population at equilibrium [30]. In presence of Ca^++^ and thiol catalysts, purified Xa5 progressively converts into native LA5 [30].

The oxidative folding of LA5 in the presence of Ca^++^ leads to the efficient formation of the native form with only a minor population of Xa5 present when the reaction is performed in the absence of thiol catalyst [30]. The reaction of LA4 in the same Ca^++^ conditions is much less efficient and the native form coexists with defined scrambled isomers, Xa4 and Xb4, even in the presence of GSSG, BME or PDI (Figs 2, 4 and 7). This behavior is reminiscent of that exhibited by the LA5 S14A and E16K mutants [30], the last mutation being linked to FH. Those two mutations disrupt the hydrogen-bonding network connecting the N and C-terminal lobes of LA5, destabilizing the domain and likely affecting its affinity for Ca^++^. We have shown recently that the conformational stability of apoLA4 is lower than that of apoLA5 [31, 32], which results in a lower affinity for the ion [19, 31]. This would explain why scrambled LA4 forms coexist with the native species at equilibrium, as observed for LA5 mutants [30]. Because FH mutants displaying lower Ca^++^ affinity cause partial retention of the full-length receptor (LDLR) in the ER or/and lower affinity for the ligand at the cell surface [36, 37] it is expected that the low folding efficiency of LA4 would be somehow linked, directly or indirectly, to a functional advantage, since otherwise selective pressure during evolution would have purged it out.

### Thermodynamic properties of LA4 native and scrambled forms

The scrambled forms appearing in the oxidative folding of LA4 (Fig 2A) trap the folding reaction. Increasing temperature destabilizes them permitting their faster reshuffling towards the more stable native state (Fig 2C). The isolated Xa4 scrambled isomer spontaneously evolves, in presence of Ca^++^ to form the natively bound isomer (Fig 8C and 8D). However, LA4 and Xa4 appear to be separated by a high-energy barrier, which makes the attainment of the native state from the scrambled form very slow and incomplete in the absence of thiols, even in the presence of high concentrations of Ca^++^ (Fig 8C1 and 8C2). This indicates that reshuffling is the rate limiting step in the LA4 oxidative folding reaction and therefore that the disulfides are well protected inside the Xa4 structure. Thiol catalysts, by lowering this energy barrier, shift the equilibrium towards the formation of LA4 in the presence of Ca^++^. Once attained, the LA4 native state is stable and does not shift into any other conformation in the absence of Ca^++^, as long as thiol catalysts are not present (Fig 8A). In the presence of BME (Fig 8B), native LA4 spontaneously reshuffles to form Xa4 and Xb4. This implies that cellular conditions weakening Ca^++^ coordination to LA4 might lead to the spontaneous reshuffling and misfolding of this LDLR module.

### LA4 and LA5 fold independently in the context of LA45

The formation of the functional LDLR upon its synthesis at the ribosome involves the formation of 30 native disulfide bonds [12]. This implies that, in principle, an enormously large number of non-native disulfide bonds between cysteines present in different domains can be formed and in fact the formation of intermediates carrying non-native disulfides has been reported at the early stages of folding of the LDLR *in vivo* [25, 26]. However, our data clearly show that no inter-domain disulfides are formed during the oxidative folding of LA45 in the presence of Ca^++^, despite the reaction flows through a large number of heterogeneous intermediates (Fig 2D). Somehow surprisingly, even when the oxidative folding was performed in the absence of Ca^++^ and in spite of the heterogeneous ensemble formed we couldn’t detect formation along the reaction of any mixed disulfide bond between cysteines corresponding to the LA4 and LA5 domains (Fig 3), which suggests that, in case they form, they have a very transient nature and the domains preferentially collapse individually. Analysis of the high mass/charge region of Fig 6A indicates that interdomain disulfides could, at most, appear in tiny amounts in the early stages of the reaction. Interestingly, when Ca^++^ was added in the 1–1000 mM range to the heterogeneous ensemble and the reaction was allowed to evolve for 48 additional hours, either in presence or absence of thiols, the distribution of species at equilibrium was almost identical to that obtained when the reaction was initiated from unfolded and reduced LA45 in presence of Ca^++^, which shows that the folding route of LA45 is fully reversible (Fig 7B and 7D). This behavior fits with the recent observation that in the ER, where Ca^++^ is abundant, the folding of the LDLR is reversible [25] and, more generally, that in the ER disulfide-containing proteins remain malleable and can be conformationally rescued [25]. The absence of inter-domain disulfides between adjacent domains during LA45 folding seems, only apparently, to contradict *in vivo* data in which inter-domain disulfides are detected at the early stages of folding [25, 26]. In fact, non-native disulfides formed between neighboring domains would only give rise to small collapses, while disulfides formed between distant domains could give rise to the observed collapse of the LDLR in the absence of native structure. The extremely large conformational search that would be imposed on the LDLR if all possible combinations of disulfides had to be searched in a rather flat potential energy surface characterized by a low content of native contacts together with our observation that two adjacent LA modules, LA4 and LA5, do not form interdomain disulfides during their oxidative folding suggest that, at least for some segments of the LDLR binding domain, the conformational search occurs as a succession of independent searches confined to individual domains.

### Does the less efficient oxidative folding of LA4 compared to LA5 serve a functional purpose?

LA4 and LA5 appear to be the key LDLR modules for extracellular lipoprotein binding and endosomal release [13, 27]. Compared to the efficient oxidative folding of LA5 [30], folding of LA4 is slower and less responsive to the presence of Ca^++^ ions as significant barriers between scrambled isomers appear to slow the reaction. It has been shown that, compared to apoLA5, apoLA4 is less stable [31] and displays a lower affinity for Ca^++^ [19, 31], which is related to its significantly slower Ca^++^ binding reaction [31]. The LDLR cycles between the membrane and the endosome where it releases the transported lipoprotein, either LDL or VLDL. Although the mechanism of lipoprotein release is not fully understood, endosomes are known to contain several ion channels whose activity rapidly lowers the endosomal pH and the Ca^++^ concentration [38, 39]. The drop in Ca^++^ concentration is sensed by both LA4 and LA5, which rapidly adjust their populations of apo and Ca^++^ bound forms to the lowering Ca^++^ endosomal concentration. As a result of Ca^++^ dissociation from LA4 and LA5 their interaction with the lipoproteins is weakened and lipoprotein release is promoted. Although both LA4 and LA5 release Ca^++^ to adjust to the changing endosomal concentrations, LA4 probably contribute most to debilitation of the LDLR/lipoprotein interface due to its lower Ca^++^ affinity [19, 31]. Given the strong structural similarity of the Ca^++^ coordination cages in native LA4 and LA5 we have proposed that the lower affinity of LA4 for Ca^++^ is related to the lower conformational stability of its apo form compared to that of LA5 [31]. The less efficient oxidative folding of LA4, compared to LA5, suggests that the lower stability of apoLA4 compared to apoLA5 is due to the presence of less stabilizing non covalent native contacts in apoLA4. The amino acid sequence of LA4 might have been selected to be particularly unstable in the absence of disulfides, the consequence being a less efficient formation of the native disulfides, a lower stability of the natively oxidized apo form and, hence, a weaker calcium binding, which would be needed to facilitate lipoprotein dissociation as soon as the endosomal calcium concentration is significantly reduced. Fortunately, although the oxidative folding of LA4 resembles that of Ca^++^ binding defective LA5 mutants (such as S14A or E16K) leading to Familial Hypercholesterolemia, in vivo folding of LA4 within LDLR does not lead to misfolding, which might be related to the assistance of chaperones such as receptor-associated protein (RAP) [34, 40] or endoplasmic reticulum DNA J domain-containing protein 5 (ERdj5), [41].
